# Prognostic Value of Systemic Inflammation Markers (NLR and Haemoglobin) in Non-Small Cell Lung Cancer: Survival Analysis from a Real-World Single-Centre Cohort Study

**DOI:** 10.3390/medicina62030467

**Published:** 2026-02-28

**Authors:** Carina Maria Golban, Lavinia Davidescu, Alexandru Alexandru, Silviu Vlad, Alina Gabriela Negru, Sorin Saftescu, Petrescu Codruta Ileana, Catalin Prodan Barbulescu, Serban Mircea Negru

**Affiliations:** 1 Doctoral School, “Victor Babes” University of Medicine and Pharmacy, Eftimie Murgu Square No. 2, 300041 Timisoara, Romania; carina.golban@umft.ro (C.M.G.); alexandru.alexandru@umft.ro (A.A.); 2 Department of Medical Disciplines, Faculty of Medicine and Pharmacy, University of Oradea, 410073 Oradea, Romania; 3 Department of Surgical Specialties, Faculty of Medicine and Pharmacy, University of Oradea, 410073 Oradea, Romania; silviu.vlad@didactic.uoradea.ro; 4 Department of Cardiology, “Victor Babes” University of Medicine and Pharmacy, Eftimie Murgu Square No. 2, 300041 Timisoara, Romania; alinanegru@umft.ro; 5 Department of Oncology, Faculty of Medicine, “Victor Babes” University of Medicine and Pharmacy Timisoara, Eftimie Murgu Square 2, 300041 Timisoara, Romania; sorin.saftescu@umft.ro (S.S.); serban.negru@umft.ro (S.M.N.); 6 Department of Anatomy and Embryology, “Victor Babes” University of Medicine and Pharmacy, 300041 Timisoara, Romania; petrescu.codruta@umft.ro (P.C.I.); catalin.prodan-barbulescu@umft.ro (C.P.B.)

**Keywords:** lung cancer, non-small cell lung cancer, NSCLC, neutrophil-to-lymphocyte

## Abstract

*Background and Objectives*: In real-world NSCLC management, prognostic assessment extends beyond tumour staging and molecular profiling, which represent a partial timeframe of disease biology. Routinely collected inflammatory and haematological markers may better reflect the dynamic host–tumour interactions during treatment. This study assessed the prognostic significance of baseline and longitudinal neutrophil-to-lymphocyte ratio (NLR) and haemoglobin levels on survival outcomes in a real-world NSCLC cohort. *Materials and Methods*: We conducted a retrospective observational cohort study of 615 patients with histologically confirmed NSCLC diagnosed between 1 May 2022 and 30 April 2024 at a tertiary referral centre in western Romania. Survival outcomes, including progression-free and overall survival, were analysed through Kaplan–Meier curves, complemented by 12-month restricted mean survival time estimates. High NLR was defined as ≥3 and low haemoglobin as <12 g/dL. Longitudinal changes were evaluated at 6 and 12 months, with 12-month analyses restricted to patients alive at that landmark. *Results*: The cohort had a median age of 66 years (IQR 60–72) and was predominantly male (66.3%). Most patients presented with advanced disease (60.3% stage IV, 23.6% stage III). At baseline, 57.1% (*n* = 351) exhibited high NLR and 39.8% (*n* = 245) had low haemoglobin. Median PFS was 9.0 months (IQR 4.5–15.5), and median OS was 16.5 months (IQR 8.5–27.0). Stage IV disease was associated with shorter PFS than stages I–II (7.0 vs. 20.8 months; log-rank *p* < 0.001). High-baseline NLR showed a borderline association with shorter PFS (adjusted HR 1.40; 95% CI 0.98–1.95). Among the 436 patients alive at 12 months, NLR increased in 56.7% of cases, and this increase showed a non-significant trend toward shorter PFS (HR 1.35; 95% CI 0.95–1.90; *p* = 0.09) in a 12-month landmark analysis. *Conclusions*: Baseline systemic inflammation and anaemia are highly prevalent in real-world NSCLC patients and cluster with advanced disease. Elevated NLR was associated with poorer survival outcomes, whereas low haemoglobin did not demonstrate a significant independent association in adjusted analyses. These haematological parameters are accessible tools for prognostic assessment in routine clinical practice.

## 1. Introduction

The management of non-small cell lung cancer (NSCLC) must continually adapt to the evolving therapeutic options while also considering the practical constraints of routine clinical care. While advances in molecular diagnostics and immuno-oncology have transformed treatment algorithms, real-world decision-making remains dependent on factors that extend beyond tumour genomics alone. This is especially true when considering that while revolutionary, biomarkers, such as genomic profiling for EGFR, ALK, or KRAS and PD-L1 expression levels, represent only a snapshot of tumour biology [1].

NSCLC remains a major global health burden, accounting for approximately 85% of the global lung cancer burden, and contributing to nearly 1.8 million deaths worldwide, even in the context of treatment advances [2,3,4]. The discrepancy between controlled clinical trial environments and routine oncology care is particularly evident in regions with limited screening uptake and high-risk populations [5,6,7]. In such settings, including much of eastern Europe, NSCLC is commonly diagnosed at advanced stages, when prognosis is poor and therapeutic choices are shaped not only by tumour characteristics but also by patient frailty, systemic inflammation, and physiological reserve [8,9]. In Romania, a combination of high smoking prevalence, environmental risk factors such as radon exposure, and limited access to organised screening programmes results in a substantial proportion of patients presenting with advanced disease [4,10,11,12].

In everyday practice, clinicians must frequently assess prognosis and treatment response using incomplete information, variable follow-up intervals, and biological signals that evolve over time rather than remaining static [13]. Patients undergo repeated clinical assessments, laboratory testing, and imaging as part of standard care, generating longitudinal data that can be leveraged in prognostic models. Systemic host-related factors—often referred to as the “macro-environment”—have emerged as important determinants of cancer outcomes. Among these, systemic inflammation and haematological status are particularly attractive in real-world oncology, as they are universally available, inexpensive, and repeatedly measured during standard care [14,15,16].

The neutrophil-to-lymphocyte ratio (NLR) reflects the balance between inflammatory activation and immune competence, two processes linked to tumour progression and treatment resistance [14,17,18,19]. Elevated NLR values, commonly defined using thresholds ranging from ≥3 to ≥5 in prior studies, have been associated with impaired anti-tumour immune response and inferior survival outcomes [20,21,22]. Similarly, haemoglobin levels serve as markers of physiological reserve and tumour-related systemic effects. Low haemoglobin levels (often defined as <12 g/dL) may reflect chronic inflammation, hypoxia, nutritional compromise, or bone marrow suppression, all of which can adversely influence therapeutic response and survival [15,23,24].

Additional biomarkers, such as EGFR, ALK, or KRAS mutations and PD-L1 expression, have revolutionised first-line therapy and strongly influence prognosis and treatment selection in NSCLC. However, these tumour-specific markers represent relatively static measurements and may not capture ongoing biological adaptation under therapeutic pressure, particularly in the context of metastatic evolution and clonal selection phenomena such as “pruning” [25,26,27,28,29,30,31,32,33,34].

Some investigations have suggested that early treatment-related changes in NLR may carry additional prognostic value, but generally the available evidence derives from selected trial populations or relatively small cohorts and focuses primarily on baseline measurements rather than longitudinal biomarker dynamics [35,36,37,38,39]. Such approaches may underestimate the clinical relevance of changes occurring during treatment and may not adequately reflect the heterogeneity of real-world patients, who frequently present with multiple comorbidities, variable performance status, and importantly, non-standardised treatment pathways due to various real-world events. For example, Matsumoto et al. reported that high-baseline NLR (≥5, as defined in their cohort) and early treatment-related increases independently predicted inferior progression-free and overall survival in advanced NSCLC receiving chemo-immunotherapy [35]. Similar findings were reported by Mandaliya et al., who observed that early post-treatment increases in NLR were associated with poorer overall survival [40]. However, these studies were limited by modest sample sizes and restricted subgroup analyses based on stage, PD-L1 status, or treatment modality.

With this context in mind, high-volume tertiary centres with real-world data records, offer a valuable opportunity to examine both baseline and dynamic prognostic biomarkers in routine oncology practice. Routinely collected longitudinal laboratory data allows evaluation of clinically meaningful endpoints within heterogeneous treatment contexts and variable follow-up intervals.

Accordingly, the present study was designed to evaluate the prognostic significance of baseline and longitudinal neutrophil-to-lymphocyte ratio and haemoglobin levels in a large, single-centre real-world cohort of patients with NSCLC. Progression-free survival was defined as the primary outcome, with overall survival as a secondary endpoint. Additionally, treatment response according to RECIST 1.1 criteria and exploratory subgroup analyses by disease stage, PD-L1 tumour proportion score, treatment modality, and molecular characteristics were performed to contextualise these findings within routine clinical practice.

## 2. Materials and Methods

### 2.1. Study Design and Population

Eligible patients were identified based on predefined criteria to ensure the consistency and completeness of clinical and laboratory data. Inclusion required age ≥ 18 years, a histologically confirmed diagnosis of NSCLC, and diagnosis within the ethics committee-approved study period. All patients were required to have baseline hematologic measurements, including neutrophil and lymphocyte counts and haemoglobin levels, as well as at least one documented follow-up visit recorded in the electronic health record. Patients with small-cell lung cancer or other non-NSCLC histologies were excluded, as were those lacking baseline laboratory data or follow-up information.

The study was conducted as a retrospective observational analysis at the Oncohelp Hospital of Timișoara, a tertiary referral centre serving approximately two million inhabitants in western Romania. Using the institutional electronic health record system, patients diagnosed with NSCLC—encompassing adenocarcinoma, squamous-cell carcinoma, and large-cell carcinoma—between 1 May 2022 and 30 April 2024, were identified. Disease staging followed the 8th edition of the TNM classification system [41]. This timeframe allowed for up to 12 months of follow-up for all patients and longer follow-up for those diagnosed earlier within the inclusion period. Patients were followed from the date of diagnosis until the administrative censoring date (30 April 2025) or last known clinical contact. Median follow-up time was estimated using the reverse Kaplan–Meier method. Progression-free survival (PFS) was defined as the time from diagnosis to radiologic or clinical disease progression according to RECIST version 1.1, or death from any cause, whichever occurred first. Patients without documented progression or death were censored at the date of last disease assessment. Overall survival (OS) was defined as the time from diagnosis to death from any cause. Patients alive at the end of the study period were censored at the date of last known contact.

To account for variable observation times and unequal risk intervals, survival analyses incorporated Restricted Mean Survival Time (RMST) at a 12-month horizon. This timeframe was selected because the majority of events occurred within the first 12 months and this timeframe provided a clinically meaningful and uniform evaluable window. Additionally, to address potential confounding by indication, Inverse Probability of Treatment Weighting (IPTW) was applied to adjust comparisons for disease stage and PD-L1 expression.

### 2.2. Data Collection

Baseline assessments occurred within one week of diagnosis, with subsequent longitudinal data gathered at 6-, 12-, 24-, and 36-month intervals. These timepoints were chosen to align with routine clinical check-ups and standard-of-care monitoring.

Demographic and clinical variables included age, sex, smoking status (pack-years), Eastern Cooperative Oncology Group (ECOG) performance status, and Charlson Comorbidity Index. Tumour-related characteristics comprised histological subtype, TNM stage, tumour size, metastatic sites, PD-L1 tumour proportion score, and molecular alterations (EGFR, ALK, ROS1, KRAS, BRAF). Baseline laboratory parameters included neutrophil and lymphocyte counts, haemoglobin, lactate dehydrogenase (LDH), and serum albumin. Longitudinal haematological measurements were collected at predefined follow-up intervals.

Treatment-related variables included therapeutic modality, treatment line, and radiologic response according to RECIST version 1.1, and treatment-related toxicity graded by CTCAE version 5.0.

High NLR was defined using a threshold of ≥3, consistent with commonly applied cutoffs in NSCLC literature and reflecting moderate systemic inflammatory burden [42,43,44]. Longitudinal increases were defined as any positive change from baseline (ΔNLR > 0).

Tumour burden and metastatic sites were evaluated at baseline, while longitudinal response (complete response, partial response, stable disease, progressive disease) was assessed at specified intervals using RECIST version 1.1. Survival endpoints (progression-free survival and overall survival) and treatment toxicity were tracked throughout the follow-up period. Data quality was maintained through double abstraction and periodic audits, yielding a Cohen’s kappa of 0.85–0.90.

### 2.3. Ethical Considerations

Ethical supervision for this retrospective analysis was obtained from the Ethics Committee of the Oncohelp Hospital in Timișoara (No. 187/04.11.2024), and all procedures were aligned with the Declaration of Helsinki. Considering the study’s retrospective nature, the Ethics Committee granted a waiver of individual informed consent, while institutional policy ensured that patients had provided broad consent for secondary data use at admission. To safeguard confidentiality, all patient records were fully de-identified prior to analysis and handled in accordance with GDPR requirements and national data protection regulations. Identifiable information remained accessible only to the designated investigators involved in data extraction, using internally generated patient identification codes.

### 2.4. Statistical Analysis

Distributional assumptions were evaluated using the Shapiro–Wilk test. Continuous variables are summarised as medians (IQR) and categorical variables as frequencies (%), with between-group comparisons performed using the Mann–Whitney U and chi-square tests, respectively. Follow-up time was estimated using the reverse Kaplan–Meier method. Progression-free and overall survival were analysed using Kaplan–Meier curves and compared with the log-rank test, with effect estimates expressed as hazard ratios and 95% confidence intervals derived from Cox regression models.

To evaluate the long-term robustness of survival findings, a secondary sensitivity analysis using RMST at 24 months was performed. This analysis was restricted to the subgroup of patients with at least 24 months of potential follow-up to ensure data maturity. Comparisons of objective response rates (ORR) accounted for treatment-by-indication bias through the application of inverse probability of treatment weighting (IPTW). Multivariable logistic regression models were used to examine associations between ORR and baseline neutrophil-to-lymphocyte ratio (NLR) or haemoglobin levels. Longitudinal changes in haematological parameters were assessed using the Mann–Whitney U test.

All statistical tests were two-sided, with a significance threshold set at *p* < 0.05. Given the low proportion of missing data, attributable to the validation procedures described above, analyses were conducted using a complete-case approach. Statistical processing was conducted using SPSS software (version 26; IBM Corp., Armonk, NY, USA).

AI Disclosure: During manuscript preparation, the authors utilised ChatGPT (OpenAI, San Francisco, CA, USA; GPT-5, December 2025 version) exclusively for linguistic refinement and readability enhancements. All scientific content, data interpretation, and statistical results remain the sole responsibility of the authors.

## 3. Results

### 3.1. Study Population—General Characteristics

The final study cohort comprised 615 patients who fulfilled all inclusion criteria. All individuals had a histologically confirmed diagnosis of NSCLC made between 1 May 2022 and 30 April 2024 and were treated and followed at the Oncohelp Hospital in Timișoara, Romania. Baseline demographic and clinical characteristics are summarised in Table 1.

At presentation, advanced disease predominated. Stage IV disease was observed in 60.3% of patients, while 23.6% were diagnosed with stage III and 16.1% with stages I–II. Metastatic involvement was documented in 371 patients (60.3%), with a median of one metastatic site (IQR 0–2). The median baseline tumour diameter measured 3.8 cm (IQR 2.1–5.8 cm). Over the course of follow-up, progression or death occurred in 63.6% of the cohort.

Histopathological assessment showed adenocarcinoma as the most frequent subtype (59.0%), followed by squamous cell carcinoma (25.7%) and other NSCLC variants (15.3%). Among adenocarcinoma cases, thyroid transcription factor-1 (TTF-1) expression was detected in 69.2%. Molecular analyses identified EGFR mutations in 14.5% of patients, ALK rearrangements in 5.5%, and KRAS mutations in 24.4%. Programmed death-ligand 1 (PD-L1) tumour proportion score distribution was <1% in 50.4%, 1–49% in 33.0%, and ≥50% in 16.6% of cases.

The cohort had a median age of 66 years (IQR 60–72), with males accounting for 66.3% of patients. A history of tobacco exposure was reported in 78.7%. The median Charlson Comorbidity Index (CCI) was 2 (IQR 1–3), and baseline Eastern Cooperative Oncology Group (ECOG) performance status had a median value of 1 (IQR 0–2), reflecting moderate functional impairment in a substantial proportion of patients. Baseline laboratory parameters demonstrated a median lactate dehydrogenase (LDH) level of 248 U/L (IQR 209–344 U/L) and a median serum albumin concentration of 34.8 g/L (IQR 29.6–39.1 g/L).

Treatment strategies varied according to disease stage and biomarker status. Chemo-immunotherapy combinations were administered to 42.3% of patients (*n* = 260), most frequently pembrolizumab combined with carboplatin and pemetrexed in advanced-stage disease. Immunotherapy monotherapy was used in 24.4% of cases (*n* = 150), commonly pembrolizumab or atezolizumab in patients with PD-L1 expression ≥ 1%. Chemotherapy and/or best supportive care was delivered to 33.3% of patients (*n* = 205), primarily in early-stage or palliative settings. Patients harbouring actionable driver mutations received targeted therapy in accordance with contemporary clinical guidelines. Patients with EGFR mutations were treated with EGFR tyrosine kinase inhibitors, most commonly Osimertinib in the first-line setting. Similarly, patients with ALK rearrangements received ALK-targeted therapy, most commonly Alectinib as first-line treatment followed by Lorlatinib upon progression.

Using the Schoenfeld approach for time-to-event outcomes, a post hoc power assessment (two-sided α = 0.05) was performed. The calculation incorporated 615 participants, 391 progression or death events, and an observed 12-month progression-free survival of 40% in the low NLR subgroup. Under these conditions, the study was adequately powered (80%) to identify a minimum hazard ratio of 1.50 for progression-free survival between patients with elevated NLR (≥3) and those with lower values.

### 3.2. Haematological Parameters at Baseline and in Evolution

At the start of the study, the cohort comprised 615 patients with NSCLC, showing a median neutrophil-to-lymphocyte ratio (NLR) of 3.2 (IQR 2.2–5.2), and more than half of the cohort (57.1%, *n* = 351) met the predefined threshold for elevated NLR (≥3). Longitudinal haematological parameters were analysed using a landmark approach. Of the 615 patients at baseline, 539 were alive at 6 months, 436 at 12 months, and 265 at 24 months. Percentages at each timepoint were calculated using the number of patients alive at the corresponding landmark as the denominator. Baseline and longitudinal haematological parameters are summarised in Table 2.

Median haemoglobin concentration at baseline was 13.9 g/dL (IQR 10.2–16.2), with low haemoglobin levels (<12 g/dL) observed in 39.8% of patients (*n* = 245). Elevated NLR was significantly more prevalent among individuals with stage IV disease compared with those in stages I–III (64.2% vs. 46.4%, χ^2^ = 19.03, *p* < 0.0001), as well as among patients with poorer performance status (ECOG 2–3 vs. 0–1: 68.6% vs. 53.4%, χ^2^ = 10.68, *p* = 0.001). Similarly, reduced haemoglobin levels were more frequently identified in stage IV disease (48.5% vs. 32.0%, χ^2^ = 16.6, *p* < 0.0001). Although low haemoglobin was numerically more common in ever-smokers than in never-smokers (42.8% vs. 38.9%), this difference did not reach statistical significance (χ^2^ = 0.61, *p* = 0.43). No significant variation in baseline NLR or haemoglobin was observed according to sex or histological subtype (*p* > 0.05).

Among the 436 patients who remained alive at 12 months, longitudinal assessment revealed a progressive increase in NLR values, with the cohort median rising from 3.2 at baseline to 4.1 (IQR 2.8–6.1) at 12 months, and 62.4% of patients (*n* = 272) meeting criteria for elevated NLR at this timepoint. The frequency of NLR elevation varied by treatment modality, occurring most commonly in patients receiving chemo-immunotherapy (67.0%), followed by chemotherapy/supportive care (62.4%) and immunotherapy alone (54.2%) (χ^2^ = 6.27, *p* = 0.043). Over the same period, haemoglobin levels declined, with the median decreasing from 13.9 g/dL at baseline to 12.1 g/dL at 12 months, and low haemoglobin levels observed in 49.8% of patients (*n* = 217). This decline was significantly more pronounced in patients with stage IV disease compared with those in earlier stages (58.1% vs. 42.9%, χ^2^ = 4.22, *p* = 0.040). In addition, increases in NLR over time were greater among patients with ECOG performance status 2–3 than among those with ECOG 0–1 (median ΔNLR +1.1 [IQR 0.3–2.4] vs. +0.5 [IQR −0.2 to +1.8], Mann–Whitney U = 2784, *p* = 0.032). Subgroup associations between haematological parameters and key clinical variables at baseline and at 12 months are summarised in Table 3.

### 3.3. Treatment Patterns and Tumour Response

Treatment exposure within the cohort was heterogeneous. Combination chemo-immunotherapy was the most frequently employed approach, administered to 260 patients (42.3%), with a median of 5 treatment cycles (IQR 3–7), most commonly involving platinum-based doublets combined with immune checkpoint inhibitors (e.g., carboplatin, pemetrexed, and pembrolizumab). Immunotherapy as monotherapy was received by 150 patients (24.4%), who completed a median of 10 cycles (IQR 6–14), predominantly with agents such as pembrolizumab or atezolizumab. The remaining 205 patients (33.3%) were managed with chemotherapy alone—most often docetaxel—or best supportive care, with a median of 4 cycles (IQR 2–6). With regard to treatment sequencing systemic therapy was delivered as first-line treatment in 454 patients (73.8%), second-line therapy in 52 patients (8.5%), and third-line or later therapy in 51 patients (8.3%). Exclusive supportive care without systemic therapy was documented in 58 cases (9.4%).

No statistically significant differences in treatment allocation were observed across sex or histological subtypes (χ^2^ test, *p* > 0.05). When expressed as proportions of the overall cohort, chemo-immunotherapy was administered to 36.1% of patients with stage IV disease compared with 6.0% of those with stages I–III (χ^2^ = 65.1, *p* < 0.001), reflecting the strong association between metastatic stage and use of combination systemic therapy. To mitigate confounding related to treatment selection when evaluating objective response rates, inverse probability of treatment weighting (IPTW) was applied; the methodological framework for this adjustment is described in detail in Section 2.4.

At 6 months, tumour response assessment in the overall cohort showed an objective response rate (ORR; complete response [CR] or partial response [PR]) of 23.4% (*n* = 144; CR 2.2%, *n* = 14; PR 21.1%, *n* = 130) and a disease control rate (DCR; CR, PR, or stable disease [SD]) of 57.8% (*n* = 356, including 34.5% SD, *n* = 212), with 42.2% of patients (*n* = 259) experiencing progressive disease (PD) within the first 6 months.

By 12 months, cumulative response rates declined, with ORR decreasing to 16.7% (*n* = 103) and DCR to 42.2% (*n* = 259), while PD had occurred in 53.3% of patients (*n* = 328). These cumulative response outcomes reflect disease progression and deaths occurring over time and are not restricted to patients alive at the 12-month landmark.

Tumour PD-L1 expression showed a strong association with treatment response. Patients with PD-L1 TPS ≥ 50% demonstrated a significantly higher ORR compared with those with TPS < 1% (33.3% vs. 14.2%, χ^2^ = 18.32, *p* < 0.001). Intermediate PD-L1 expression (TPS 1–49%) was associated with an ORR of 23.2%. Baseline inflammatory and haematological parameters were also associated with response patterns. Patients with high-baseline NLR (≥3) exhibited lower disease control rates compared with those with NLR < 3 (49.9% vs. 67.8%, χ^2^ = 22.26, *p* < 0.001), while low haemoglobin levels (<12 g/dL) were associated with a higher frequency of PD (49.5% vs. 36.8%, χ^2^ = 9.97, *p* = 0.002).

Disease stage had a measurable impact on tumour response. Patients with stage IV disease had a significantly lower ORR compared with those with stages I–III combined (19.4% vs. 32.8%, χ^2^ = 14.16, *p* < 0.001). With respect to treatment modality, chemo-immunotherapy was associated with superior response rates compared with immunotherapy alone (27.3% vs. 16.7%, χ^2^ =6.01, *p* = 0.014). Tumour response outcomes stratified by treatment type, PD-L1 TPS, and disease stage are summarised in Table 4.

### 3.4. Survival Outcomes

Survival analyses were performed using the full baseline cohort of 615 patients, with censoring applied according to last follow-up. Across the entire study population (*n* = 615), median progression-free survival (PFS) was 9.0 months (IQR 4.5–15.5), with a 12-month restricted mean survival time (RMST) of 7.2 months. Median overall survival (OS) was 16.5 months (IQR 8.5–27.0), corresponding to a 12-month OS-RMST of 10.5 months.

**Figure 1:**
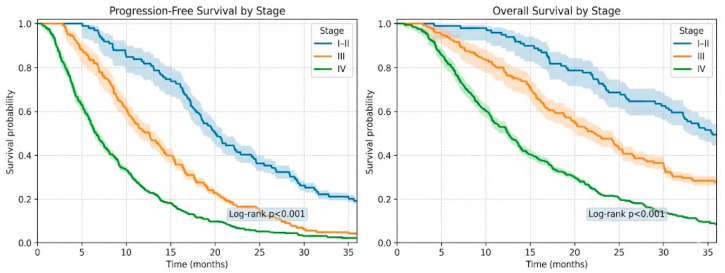
Stage-stratified Kaplan–Meier of progression-free survival and overall survival in NSCLC.

Survival outcomes demonstrated a clear stage-dependent gradient. Patients with stage IV disease (*n* = 371) had a median PFS of 7.0 months (IQR 3.8–12.2), compared with 12.5 months (IQR 6.9–18.3) in stage III (*n* = 145) and 20.8 months (IQR 12.4–28.6) in stages I–II (*n* = 99) (log-rank *p* < 0.001). A similar pattern was observed for OS, with median survival of 12.0 months in stage IV, 24.2 months in stage III, and 35.9 months in stages I–II (log-rank *p* < 0.001). Stage-stratified Kaplan–Meier curves are presented in Figure 1.

To evaluate the independent prognostic impact of haematological parameters, multivariable Cox regression models were constructed adjusting for age, sex, disease stage, ECOG performance status, smoking history, PD-L1 tumour proportion score, and treatment-start delay.

Elevated baseline neutrophil-to-lymphocyte ratio (NLR ≥ 3) was associated with a 40% higher hazard of progression (HR 1.40, 95% CI 0.98–1.95) and a 25% higher hazard of mortality (HR 1.25, 95% CI 0.90–1.75), although these associations did not reach conventional statistical significance. Consistent with this pattern, disease control at 6 months was achieved less frequently in patients with NLR ≥ 3 compared with those with lower values (49.9% vs. 67.8%, *p* < 0.001).

Longitudinal inflammatory dynamics further refined risk stratification. An increase in NLR over time, observed in 56.7% of patients alive at 12 months, was associated with an increased hazard of progression (HR 1.35, 95% CI 0.95–1.90) and overall survival (HR 1.20, 95% CI 0.85–1.70) but neither reaching significance (see Table 5).

In contrast, haemoglobin dynamics showed weaker associations with time-to-event outcomes. Although haemoglobin levels declined over follow-up and low haemoglobin (<12 g/dL) was common at 12 months, these changes were not significantly associated with either progression-free survival (HR 1.10, 95% CI 0.80–1.50) or overall survival (HR 1.05, 95% CI 0.75–1.45).

All multivariable survival analyses are summarised in Table 5.

### 3.5. Subgroup Analyses

Subgroup analyses were impacted by the cohort’s PD-L1 distribution. Given the prevalence of PD-L1-negative tumours (50.4%, *n* = 310) and the lower prevalence of high expressors (16.6%, *n* = 102), the comparison of PD-L1 ≥ 50% versus <1% is reflective of the non-selected, low-PDL1 population; The comparison of first-line chemo-immunotherapy versus immunotherapy alone cannot be directly interpreted in this cohort, as treatment selection is heavily confounded by PD-L1 status in clinical practice.

Subgroup analyses revealed modified survival patterns. Patients with PD-L1 TPS ≥ 50% (*n* = 102) had longer PFS (median 9.4 months, IQR 4.8–14.5) and OS (15.0 months, IQR 8.5–26.0) compared to those with TPS < 1% (*n* = 310; PFS 6.5 months, IQR 3.4–12.1; OS 11.2 months, IQR 5.8–19.4; log-rank *p* = 0.02). A direct, unadjusted comparison between treatment types was not performed due to significant confounding by the PD-L1 distribution.

Survival outcomes varied substantially according to baseline functional status. Patients with preserved performance status (ECOG 0–1; *n* = 468, 76.1%) experienced a median progression-free survival (PFS) of 10.4 months (IQR 5.8–17.8), with a 12-month restricted mean survival time (RMST) for PFS of 7.3 months. Median overall survival (OS) in this group reached 19.2 months (IQR 11.4–31.6), corresponding to a 12-month OS-RMST of 13.2 months. By comparison, individuals with ECOG 2–3 (*n* = 147, 23.9%) had shorter outcomes, with a median PFS of 6.5 months (IQR 3.3–11.3) and a 12-month PFS-RMST of 5.0 months. Median OS in this subgroup was 12.6 months (IQR 6.5–20.4), and the 12-month OS-RMST was 8.9 months. Given that ECOG performance status was included as a covariate in the multivariable Cox regression models, no additional hypothesis testing was undertaken for this comparison.

Within the subgroup of patients harbouring EGFR mutations (*n* = 89), all of whom received osimertinib, survival outcomes were superior to those observed in EGFR wild-type disease. Median PFS was 12.5 months (IQR 6.9–17.2) in EGFR-mutant patients, compared with 9.4 months (IQR 4.6–16.0) in the wild-type population (log-rank *p* = 0.032). A similar pattern was observed for overall survival, with median OS of 25.4 months (IQR 15.1–32.3) in the EGFR-mutant group versus 16.5 months (IQR 8.6–27.7) among wild-type patients (log-rank *p* = 0.047), consistent with the benefit of targeted therapy.

Descriptive analyses of patients with ALK rearrangements (*n* = 34) demonstrated favourable survival outcomes. Median PFS in this subgroup was 19.8 months (IQR 14.8–25.4), while median OS reached 35.5 months (IQR 29.0–45.9). At 12 months, RMST values were 10.3 months for PFS and 11.4 months for OS.

Within stage IV non-EGFR/ALK-altered disease (*n* = 193), PD-L1 tumour proportion score retained prognostic value independent of the absence of targeted therapeutic options. Patients with PD-L1 TPS < 1% (*n* = 79) had a median PFS of 7.4 months (IQR 3.7–12.9) and a median OS of 13.4 months (IQR 6.7–22.3). For those with TPS 1–49% (*n* = 31), median PFS and OS were 9.3 months (IQR 4.6–15.8) and 13.5 months (IQR 8.6–27.1), respectively. Patients with TPS ≥ 50% (*n* = 83) achieved the longest outcomes within this subgroup, with a median PFS of 10.2 months (IQR 5.1–15.5) and a median OS of 16.7 months (IQR 9.2–27.8).

Survival outcomes are summarised in Table 6.

## 4. Discussion

This retrospective real-world cohort study evaluated the prognostic relevance of baseline and longitudinal haematological biomarkers—primarily NLR and haemoglobin—in 615 patients with histologically confirmed NSCLC treated at the Oncohelp Hospital in Timișoara, Romania. The cohort reflects a referral centre population with a high burden of advanced disease: 60.3% of patients were stage IV and 23.6% stage III, with substantial comorbidity (median CCI 2) and functional heterogeneity (23.9% ECOG 2–3). In this context, we aimed to characterise how readily available inflammatory and anaemia-related markers behave over time and how they align with key clinical features, treatment selection, and survival patterns typical of routine oncology care.

A central observation of this study is that elevated systemic inflammation and anaemia were common at baseline and clustered with more advanced disease. High-baseline NLR (≥3) affected 57.1% of patients and was more frequent in stage IV than in stages I–III, consistent with the concept that tumour burden and metastatic spread are associated with a more pronounced inflammatory phenotype [20,45]. Similarly, low haemoglobin (<12 g/dL) was common (39.8%) and more frequent among stage IV patients, reflecting a combination of cancer-related inflammation, nutritional status, marrow reserve, and comorbidity burden [46]. These cross-sectional patterns are clinically intuitive and mirror daily practice in referral centres, where patients often present late (especially in the context of developing countries), after symptom escalation, or following prior diagnostic delays [47,48]. Although sampling variability was not directly evaluated in our cohort, prior studies have demonstrated that intratumoral heterogeneity and specimen type (small biopsy versus surgical resection) may contribute to discordant PD-L1 tumour proportion score classification [49]. Differences in sampling may represent a plausible explanation for variability observed in real-world datasets.

A notable characteristic of our cohort was the preservation of functional status despite a heavy burden of risk factors and comorbidities. Although 78.7% of patients were ever-smokers and the median Charlson Comorbidity Index (CCI) was 2, the median ECOG performance status remained 1 (IQR 0–2). This dissociation between high disease burden—evidenced by the predominance of Stage IV diagnoses—and retained physical independence offers a critical insight into the healthcare-seeking behaviour of this population.

The data suggests that patients likely engage in “symptom normalisation”, attributing early warning signs (e.g., cough, dyspnoea) to their history of smoking or chronic conditions like hypertension and chronic obstructive pulmonary disease (COPD) rather than a new malignancy. Consequently, they delay seeking medical help until symptoms become clinically undeniable. However, importantly, this presentation occurs before the disease significantly erodes their daily functionality. Unlike populations that present in terminal decline, these patients seek care in a window where the tumour burden is high, yet they remain physically fit enough to tolerate systemic therapies. This pattern highlights a critical missed opportunity for early detection, as the preservation of “day-to-day” function may paradoxically reinforce the patient’s decision to postpone medical evaluation until the disease is advanced. Such patterns are supported by published literature and are in accordance with our previous research [50,51,52].

Longitudinally, NLR increased over follow-up, and this increase appeared more frequently in patients receiving chemo-immunotherapy than in those receiving immunotherapy alone. In a real-world setting, this pattern can plausibly reflect multiple overlapping mechanisms: evolving disease biology, treatment-related effects (including corticosteroids and intercurrent infections), and the confounding effect of indication (patients selected for combination treatment may have a different disease profile than those selected for monotherapy). Importantly, the observed increase in NLR over time is directionally concordant with the notion that worsening inflammatory balance often accompanies progression and declining clinical status [43,53].

Treatment patterns in the cohort were consistent with current systemic therapy paradigms in NSCLC. Overall, chemo-immunotherapy was the most common treatment category (42.3%), followed by immunotherapy alone (24.4%) and chemotherapy/supportive care (33.3%). A key real-world finding was the strong association between stage and treatment selection: stage IV patients were substantially more likely to receive chemo-immunotherapy than patients with stages I–III disease (36.1% vs. 6.0%, χ^2^ = 65.1, *p* < 0.001). Perioperative chemo-immunotherapy in early-stage NSCLC represents a relatively recent therapeutic development, and national health technology assessment and reimbursement implementation in Romania occurred in a stepwise manner [54]. Consequently, limited uptake in earlier-stage disease during the study window is probably related to access and implementation rather than purely stage-driven clinical decision-making. This pattern remains directionally consistent with broader treatment paradigms in which metastatic disease has historically been the primary setting for systemic combination strategies [55]. Overall, the observed predominance of chemo-immunotherapy in stage IV disease appears to be influenced more strongly by regulatory timing and access dynamics than by theoretical stage-based treatment preferences, although real-world cohorts typically include broader patient condition profiles compared to clinical trials [56,57].

Survival estimates in this cohort followed expected gradients by stage. Stage IV patients had substantially shorter median PFS and OS than stage III and stages I–II, which is consistent with both clinical trial control arms and real-world series showing inferior outcomes in metastatic disease, particularly in heterogeneous populations with comorbidities and variable performance status. Trial benchmarks, such as KEYNOTE-189 (pembrolizumab plus platinum/pemetrexed in metastatic non-squamous NSCLC) and KEYNOTE-024 (pembrolizumab monotherapy in PD-L1 ≥ 50%) [36,38], contextualise the survival range typically observed in advanced disease under immunotherapy-based strategies. The improved outcomes among patients with higher PD-L1 expression in our data are also directionally concordant with the predictive role of PD-L1 in immunotherapy-treated populations described in the established literature [26].

The distribution of PD-L1 tumour proportion score (TPS) categories in our cohort differs quantitatively from some previously published national series. harmonisation studies have demonstrated that different immunohistochemical assays are not fully interchangeable [58,59].

The survival outcomes for the ALK rearrangement-positive subgroup, which demonstrated a median PFS of 19.8 months and a median OS of 35.5 months, must be interpreted with caution. These results are derived from a reduced sample size of only 34 participants. A small cohort size increases the risk of imprecision in descriptive metrics like the 12-month RMST for PFS (10.3 months) and OS (11.4 months), highlighting the need for validation in larger, prospective, and ideally multicentric studies.

A practical strength of NLR and haemoglobin is their accessibility and low cost, making them attractive for routine risk stratification in settings where advanced molecular or immunologic profiling may not be uniformly available [60]. In a referral centre cohort with a high proportion of stage IV disease and substantial comorbidity, simple blood-based markers may help identify patients who warrant closer monitoring, earlier supportive care integration, or more intensive follow-up scheduling. However, these markers are nonspecific and can be influenced by infection, corticosteroids, marrow suppression, and other comorbid conditions; therefore, they should be interpreted as part of a broader clinical and oncologic assessment rather than as stand-alone decision tools.

This study has limitations inherent to retrospective, single-centre real-world datasets. Treatment selection is strongly confounded by indication (e.g., stage, PD-L1, performance status), limiting causal inference from unadjusted comparisons across treatment categories. Response assessment is incompletely captured in structured form, and unstructured response proxies likely underestimate true response rates and have variable timing.

This study did not perform subgroup analysis of patients treated with surgical resection or radiotherapy to avoid the added complexity of local modalities that primarily reduce tumour burden and introduce variables such as sequencing and timing with systemic therapy, without substantially altering core PD-L1 expression or tumour–immune interactions relevant to systemic response; the key exception is early-stage cases cured by complete (R0) resection alone, where PD-L1 has negligible predictive value due to absent residual disease. The analysis focused on contexts of persistent tumour burden and molecular interactions.

Longitudinal laboratory availability decreases over time due to progression and mortality, which may introduce survivorship bias in later timepoints. Additionally, supportive medications (e.g., corticosteroids, G-CSF) and intercurrent infections—factors that can materially affect neutrophil and lymphocyte counts—are not systematically captured, which may dilute associations between inflammatory markers and outcome.

Future work should prioritise (1) explicit anchoring of time-to-event analyses to treatment initiation when feasible, (2) improved capture or extraction of imaging responses (radiology reports, structured RECIST variables), enabling more credible ORR/DCR analyses, and (3) adjusted comparative effectiveness analyses (e.g., IPTW or multivariable methods) within better-defined subcohorts (e.g., metastatic EGFR/ALK-negative patients, or PD-L1 ≥ 50% for monotherapy comparability). Prospective, multicentre validation in Romanian and regional populations would further strengthen generalizability and help define pragmatic cutoffs and monitoring schedules for NLR and anaemia-based risk stratification in routine NSCLC care.

## 5. Conclusions

In this real-world cohort of 615 patients with NSCLC, outcomes reflected a referral centre population with a high burden of advanced disease and comorbidity. Stage remained the dominant prognostic factor, with substantially shorter progression-free and overall survival observed in patients with stage IV disease compared with earlier stages.

Baseline systemic inflammation and anaemia were common and clinically meaningful. Elevated neutrophil-to-lymphocyte ratio (NLR ≥ 3) and low haemoglobin (<12 g/dL) clustered with advanced stage and poorer functional status, and longitudinal increases in NLR further identified patients at higher risk of adverse outcomes. These readily available biomarkers may support pragmatic risk stratification in routine clinical practice.

Treatment allocation patterns were consistent with contemporary standards, with chemo-immunotherapy used predominantly in metastatic disease and targeted therapies conferring expected survival benefits in molecularly defined subgroups. Data availability highlights the need for cross-institutionally homogenous routine electronic health record data to facilitate patients journey traceability.

Overall, this study supports the value of simple haematological markers for prognostic assessment in real-world NSCLC populations and underscores the need for improved response capture and adjusted analyses to better evaluate treatment effectiveness outside clinical trials.

## Figures and Tables

**Table 1:** Baseline demographic and clinical characteristics of the study population. IQR indicates the interquartile range (25th–75th percentile). Abbreviations: NSCLC, non-small cell lung cancer; ECOG, Eastern Cooperative Oncology Group; LDH, lactate dehydrogenase.

Variable	Value
Age, years, median (IQR)	66 (60–72)
Sex, *n* (%)	
Male Female	408 (66.3) 207 (33.7)
Histology, *n* (%)	
Adenocarcinoma Squamous cell carcinoma Other NSCLC	363 (59.0%) 158 (25.7%) 94 (15.3%)
Stage, *n* (%)	
I–II III IV	99 (16.1%) 145 (23.6%) 371 (60.3%)
Smoking Status, *n* (%)	
Smoker Non-smoker	484 (78.7%) 131 (21.3%)
Charlson Comorbidity Index, median (IQR)	2 (1–3)
ECOG Status, *n* (%)	
0–1 2–3	468 (76.1%) 147 (23.9%)
Tumour size (cm), median (IQR)	3.8 (2.1–5.8)
LDH, U/L, median (IQR) Albumin, g/L, median (IQR)	248 (209–344) 34.8 (29.6–39.1)
Metastatic Sites, *n* (%)	
0 ≥1	244 (39.7%) 371 (60.3%)
Treatment, *n* (%)	
Chemo-immunotherapy Immunotherapy alone Chemotherapy/Supportive care	260 (42.3%) 150 (24.4%) 205 (33.3%)
Line of therapy, *n* (%)	
First-line systemic therapy Second-line therapy Third-line therapy Exclusive supportive care	454 (73.8%) 52 (8.5%) 51(8.3%) 58 (9.4%)
Median treatment cycles (IQR)	
Chemo-immunotherapy Immunotherapy alone Chemotherapy	5 (3–7) 10 (6–14) 4 (2–6)
PD-L1 TPS, *n* (%)	
<1% 1–49% ≥50%	310 (50.4%) 203 (33.0%) 102 (16.6%)
Molecular Markers, *n* (%)	
EGFR ALK KRAS	89 (14.5%) 34 (5.5%) 150 (24.4%)
Significant Event (Progression/Death), *n* (%)	391 (63.6%)

**Table 2:** Longitudinal Assessment of Haematological Parameters. NLR = Neutrophil-to-Lymphocyte Ratio.

Parameter	Baseline	6 Months	12 Months	24 Months
NLR, median (IQR)	3.2 (2.2–5.2)	3.7 (2.5–5.6)	4.1 (2.8–6.1)	4.3 (3.0–6.4)
High NLR (≥3), *n* (%)	351 (57.1%)	329 (61.0%)	272 (62.4%)	169 (63.8%)
Haemoglobin, g/dL, median (IQR)	13.9 (10.2–16.2)	13.4 (10.3–13.8)	12.1 (9.8–13.2)	11.5 (9.4–12.2)
Low Haemoglobin (<12 g/dL), *n* (%)	245 (39.8%)	233 (43.2%)	217 (49.8%)	149 (56.2%)

**Table 3:** Associations Between Haematological Parameters and Clinical Characteristics at Baseline and at 12 Months.

Clinical Variable	High NLR ≥ 3, *n* (%)	*p*-Value	Low Haemoglobin < 12 g/dL, *n* (%)	*p*-Value
Stage IV (*n* = 371)	238 (64.2%)	<0.0001	180 (48.5%)	<0.0001
Stages I–III (*n* = 244)	113 (46.4%)		78 (32.0%)	-
ECOG 2–3 (*n* = 147)	101 (68.6%)	0.001		-
ECOG 0–1 (*n* = 468)	250 (53.4%)			
**Twelve-Month Longitudinal Associations (Among Patients Alive at 12 Months, *n* = 436)**				
**High NLR (≥3) by Treatment Modality**				
Treatment Modality	High NLR ≥ 3, %		*p*-value	
Chemo-immunotherapy	67.0%			
Chemotherapy/Supportive care	62.4%			
Immunotherapy alone	54.2%		0.043	
**Change in NLR (ΔNLR) by ECOG Status**				
ECOG Status	Median ΔNLR (IQR)		*p*-value	
ECOG 2–3	+1.1 (0.3–2.4)			
ECOG 0–1	+0.5 (−0.2–1.8)		0.032	

**Table 4:** Tumour Response at 6 Months by Treatment Type, PD-L1 TPS, and Stage.

Characteristic	CR, *n* (%)	PR, *n* (%)	SD, *n* (%)	PD, *n* (%)	ORR (CR + PR), *n* (%)	DCR (CR + PR + SD), *n* (%)
Overall (*n* = 615)	14 (2.3%)	130 (21.1%)	212 (34.5%)	259 (42.1%)	144 (23.4%)	356 (57.8%)
Treatment Type						
Chemo-immunotherapy (*n* = 260)	7 (2.7%)	64 (24.6%)	88 (33.8%)	101 (38.8%)	71 (27.3%)	159 (61.2%)
Immunotherapy alone (*n* = 150)	3 (2.0%)	22 (14.7%)	53 (35.3%)	72 (48.0%)	25 (16.7%)	78 (52.0%)
Chemo/Supportive care (*n* = 205)	2 (1.0%)	37 (18.0%)	71 (34.6%)	95 (46.3%)	39 (19.0%)	110 (53.7%)
PD-L1 TPS						
<1% (*n* = 310)	7 (2.3%)	36 (11.6%)	105 (33.9%)	162 (52.3%)	44 (14.2%)	153 (49.4%)
1–49% (*n* = 203)	3 (1.5%)	44 (21.7%)	71 (35.0%)	85 (41.9%)	47 (23.2%)	118 (58.1%)
≥50% (*n* = 102)	4 (3.9%)	30 (29.4%)	34 (33.3%)	34 (33.3%)	34 (33.3%)	68 (66.7%)
Stage						
I–II (*n* = 99)	6 (6.1%)	29 (29.3%)	41 (41.4%)	23 (23.2%)	35 (35.4%)	76 (76.8%)
III (*n* = 145)	4 (2.8%)	41 (28.3%)	52 (35.9%)	48 (33.1%)	45 (31.0%)	97 (66.9%)
IV (*n* = 371)	6 (1.6%)	66 (17.8%)	123 (33.2%)	176 (47.4%)	72 (19.4%)	195 (52.6%)

ORR = Objective Response Rate; CR = Complete Response; PR = Partial Response; SD = Stable Disease; PD = Progressive Disease; DCR = Disease Control Rate.

**Table 5:** Multivariable Cox Regression Analysis of Haematological Parameters and Survival Outcomes.

Variable	PFS HR (95% CI)	*p*-Value	OS HR (95% CI)	*p*-Value
NLR ≥ 3 (baseline)	1.40 (0.98–1.95)	0.07	1.25 (0.90–1.75)	0.13
Increase in NLR (ΔNLR > 0)	1.35 (0.95–1.90)	0.09	1.20 (0.85–1.70)	0.21
Low haemoglobin (<12 g/dL)	1.10 (0.80–1.50)	0.45	1.05 (0.75–1.45)	0.46

**Table 6:** Median Progression-Free and Overall Survival According to Clinico-pathological and Molecular Features.

Characteristic	PFS, Median (IQR), Months	OS, Median (IQR), Months	PFS RMST at 12 Months, Months	OS RMST at 12 Months, Months
Overall	9.0 (4.5–15.5)	16.5 (8.5–27.0)	7.2	10.5
Stage				
I–II (*n* = 99)	20.8	35.9	10.2	11.8
III (*n* = 145)	12.5	24.2	8.5	11.0
IV (*n* = 371)	7.0	12.0	6.2	8.8
PD-L1 TPS				
<1% (*n* = 310)	6.5 (3.4–12.1)	11.2 (5.8–19.4)	6.5	9.3
1–49% (*n* = 203)	8.4 (4.2–14.9)	14.8 (7.8–25.2)	7.5	10.4
≥50% (*n* = 102)	9.4 (4.8–14.5)	15.0 (8.5–26.0)	7.8	10.8
Stage IV without EGFR/ALK alterations				
TPS <1% (*n* = 79)	7.4 (3.7–12.9)	13.4 (6.7–22.3)	5.3	8.2
TPS 1–49% (*n* = 31)	9.3 (4.6–15.8)	13.5 (8.6–27.1)	6.6	9.2
TPS ≥50% (*n* = 83)	10.2 (5.1–15.5)	16.7 (9.2–27.8)	7.4	9.9
Molecular Markers				
EGFR+ (*n* = 89)	12.5 (6.9–17.2)	25.4 (15.1–32.3)	9.2	11.2
EGFR wild-type (*n* = 526)	9.4 (4.6–16.0)	16.5 (8.6–27.7)	7.5	10.5
ALK+ (*n* = 34)	19.8 (14.8–25.4)	35.5 (29.0–45.9)	10.3	11.4
ECOG 0–1 (*n* = 468)	10.4 (5.8–17.8)	19.2 (11.4–31.6)	7.3	13.2
ECOG 2–3 (*n* = 147)	6.5 (3.3–11.3)	12.6 (6.5–20.4)	5.0	8.9

## Data Availability

The data presented in this study are available on request from the corresponding author. The data are not publicly available due to legal and ethical considerations.

## Abbreviations

Abbreviations used in the manuscript:

NSCLC	Non-Small Cell Lung Cancer
NLR	Neutrophil-to-Lymphocyte Ratio
IQR	Interquartile Range
PFS	Progression-Free Survival
OS	Overall Survival
HR	Hazard Ratio
CI	Confidence Interval
RMST	Restricted Mean Survival Time
RECIST	Response Evaluation Criteria in Solid Tumours
TNM	Tumour–Node–Metastasis Classification
IPTW	Inverse Probability of Treatment Weighting
PD-L1	Programmed Death-Ligand 1
TPS	Tumour Proportion Score
EGFR	Epidermal Growth Factor Receptor
ALK	Anaplastic Lymphoma Kinase
KRAS	Kirsten Rat Sarcoma Viral Oncogene Homologue
ROS1	ROS Proto-Oncogene 1, Receptor Tyrosine Kinase
BRAF	B-Raf Proto-Oncogene, Serine/Threonine Kinase
LDH	Lactate Dehydrogenase
ECOG	Eastern Cooperative Oncology Group
CCI	Charlson Comorbidity Index
ORR	Objective Response Rate
DCR	Disease Control Rate
CR	Complete Response
PR	Partial Response
SD	Stable Disease
PD	Progressive Disease
CTCAE	Common Terminology Criteria for Adverse Events
GDPR	General Data Protection Regulation
SPSS	Statistical Package for the Social Sciences
TTF-1	Thyroid Transcription Factor-1
